# Enhancement of neutrophil chemotaxis by *trans*-anethole-treated *Staphylococcus aureus* strains

**DOI:** 10.1371/journal.pone.0284042

**Published:** 2023-04-07

**Authors:** Paweł Kwiatkowski, Aleksandra Tabiś, Peter Sobolewski, Wojciech Płaziński, Agata Pruss, Monika Sienkiewicz, Barbara Dołęgowska, Iwona Wojciechowska-Koszko

**Affiliations:** 1 Department of Diagnostic Immunology, Pomeranian Medical University in Szczecin, Szczecin, Poland; 2 Department of Food Hygiene and Consumer Health Protection, Wroclaw University of Environmental and Life Sciences, Wroclaw, Poland; 3 Department of Polymer and Biomaterials Science, Faculty of Chemical Technology and Engineering, West Pomeranian University of Technology, Szczecin, Poland; 4 Jerzy Haber Institute of Catalysis and Surface Chemistry, Polish Academy of Sciences, Krakow, Poland; 5 Department of Biopharmacy, Medical University of Lublin, Lublin, Poland; 6 Department of Laboratory Medicine, Pomeranian Medical University in Szczecin, Szczecin, Poland; 7 Department of Pharmaceutical Microbiology and Microbiological Diagnostic, Medical University of Lodz, Łódź, Poland; University of Auckland, NEW ZEALAND

## Abstract

This study aimed to analyze the chemotactic response of differentiated HL-60 neutrophil-like (dHL-60) cells to *trans*-anethole (TA)-treated *Staphylococcus aureus* strains. Special attention was paid to evaluate the influence of TA on the *chp* gene expression level, as well as molecular docking and molecular dynamics (MD) simulation studies on interactions of TA with chemotaxis inhibitory protein of *S*. *aureus* (CHIPS). The following parameters were studied: susceptibility to TA using the agar diffusion method, the *chp* gene detection and its expression under TA influence, and clonal diversity of *S*. *aureus* strains using molecular techniques. Furthermore, a chemotactic response of dHL-60 cells to TA-treated *S*. *aureus* using Boyden chamber assay was detected and molecular modeling using both the docking methodology and unbiased MD simulations was conducted. It was found that TA showed antibacterial activity against all strains. Three genotypes and one unique pattern were distinguished among the strains. 50% of the isolates were *chp*-positive. It was observed that TA reduced/inhibited *chp* gene expression in most *S*. *aureus* strains. Enhanced chemotactic response of dHL-60 cells to TA-treated *S*. *aureus* strains was also noted. This correlation was similar for both *chp*-positive and *chp*-negative strains. Both molecular docking and MD simulations studies confirmed that TA is preferentially bound in the complement component 5a/CHIPS interface interaction region and can interfere with any processes exploiting this binding cavity. It has been proven that dHL-60 cells exhibited a higher chemotactic response to TA-treated *S*. *aureus* strains in comparison to non-treated bacteria, regardless of the achieved expression of the *chp* gene or its lack. Nevertheless, further analyses are required to understand this mechanism better.

## Introduction

Chemotaxis is fundamental for active movement of cells towards an external gradient of chemicals. This phenomenon plays a pivotal role in many primary human physiological processes, such as trafficking of lymphocytes, recruitment of leukocytes to the site of inflammation, and patterning neuronal cells [1]. Up to date, numerous chemoattractants have been discovered, including formylated peptides (e.g., N-formyl-L-methionyl-L-leucyl-phenylalanine known as fMLP), leukotriene B4, platelet-activating factor, chemokines (e.g., interleukin-8 –IL-8 known as CXCL8) and released through subsequent cascades of complement system reactions–complement components 3a and 5a (C5a) [2–4]. All chemoattractants mentioned above bind to target immunocompetent cells via specific receptors belonging to the G protein-coupled receptor superfamily [5].

*Staphylococcus aureus* is a pathogen responsible for a great variety of human diseases. It is characterized with an extensive range of virulence factors that influence the innate and adaptive immune response [6, 7]. Such factors include microbial surface components recognizing adhesive matrix molecules, such as chemotaxis inhibitory protein of *S*. *aureus* (CHIPS)–an exoprotein (molecular weight– 14.1 kDa) encoded by the *chp* gene and located with other virulence genes (*sea*/*sep*, *sak*, *scn*) within the immune evasion cluster (located on a mobile genetic element known as βC-Фs) [8–11]. It is estimated that >60% of *S*. *aureus* strains can produce CHIPS [3, 10, 12]. It exhibits an inhibitory effect on the chemotaxis and activation processes of neutrophils and monocytes, which move to the site of inflammatory reaction under the influence of chemotactic factors, e.g., C5a or fMLP [10]. During the inflammatory response, chemoattractants target host cells by binding and activating C5a (C5aR) and formylated peptide (FPR) receptors. The primary function of CHIPS is to block these receptors by directly binding them through a calcium ion-dependent pathway, leading to an impaired phagocyte response [3, 8].

Use of natural chemical compounds, including essential oils (EOs) or their main components, to stimulate the immune system to fight pathogens has becoming increasingly common. Besides, over the past decade, there have been more and more efforts to understand their mechanism of action [13]. For instance, in our previous study, we observed that *S*. *aureus* ATCC 25904 (Newman) pre-cultured on medium supplemented with subinhibitory concentration of *trans*-anethole (TA–compound occurs naturally in some plants and is obtained, e.g., from the fennel, anise, or star anise EOs) and then incorporated to the blood of healthy volunteers, which resulted, i.e. in increased level of chemotactic cytokine (IL-8) production compared to non-treated cells [14]. Hence, this study aimed to analyze the chemotactic response of differentiated HL-60 neutrophil-like (dHL-60) cells to TA-treated *S*. *aureus* strains. Special attention was paid to evaluate the influence of TA on the *chp* gene expression level, as well as molecular docking and molecular dynamics (MD) simulation studies of TA interactions with CHIPS. Furthermore, we wanted to determine the chemotactic response of dHL-60 cells to *chp*-positive and *chp*-negative TA-treated *S*. *aureus* strains, including their clonal diversity. To the authors’ best knowledge, this is the first study which implements such approach towards the possible impact of TA-treated *S*. *aureus* on neutrophil chemotaxis, including *chp* expression under TA influence and *in silico* molecular modeling.

## Materials and methods

### Strains and culture conditions

Twenty *S*. *aureus* strains, belonging to the Department of Microbiology, Immunology, and Laboratory Medicine of the Pomeranian Medical University in Szczecin collection, isolated from nasal carriers in a population of healthy adults, were used in this study. All samples were cultured on Columbia agar supplemented with 5% sheep blood (bioMérieux, Warsaw, Poland) and incubated overnight at 37°C in aerobic atmosphere. The strains were identified by biochemical test GP Vitek 2 Compact (bioMérieux, Warsaw, Poland) and all of them harbored the *nuc* gene. All strains were susceptible to methicillin (MSSA) with MIC ≤0.5 mg/l and ≤1.5 mg/l, respectively, for oxacillin and cefoxitin (see S1 Table in S1 File). *S*. *aureus* ATCC 25904 (Newman) and *S*. *aureus* ATCC 6538 were used as control strains.

### Clonal diversity of *S*. *aureus* strains

To demonstrate the clonal diversity of the analyzed *S*. *aureus* strains, they were subjected to molecular typing by pulsed-field gel electrophoresis (PFGE), using CHEF Bacterial Genomic DNA Plug Kits (Bio-Rad, Marnes-la-Coquette, France) according to the procedure described by Masiuk et al. [15]. Briefly, digestion of whole-genomic DNA was performed using the *SmaI* enzyme (MBI Fermentas, Ontario, Canada) according to the manufacturer’s protocol. PFGE was conducted using a CHEF DR III apparatus (Bio-Rad, Marnes-la-Coquette, France) in 2% agarose gel (DNA Gdansk, Gdansk, Poland) and 1×TBE (Tris-borate-EDTA) buffer with specific parameters of electrophoresis described by Masiuk et al. [15]. After electrophoresis, the gel was stained with ethidium bromide in a covered container for 30 min, visualized and photographed using a GelDoc-It2 Imager gel imaging system (Analytik Jena US LLC, Upland, CA, USA). *S*. *aureus* ATCC 25904 and *S*. *aureus* ATCC 6538 strains were included in the study.

The PFGE macrorestriction profiles were analyzed using FPQuest software (Bio-Rad, Marnes-la-Coquette, France). Classification of individual restriction patterns for each genetic profile was conducted using UPGMA (Unweighted Pair Group Method with Arithmetic Mean) and Dice’s coefficient (2.0%). PFGE results were presented as a dendrogram.

### Assessment of subinhibitory concentration of TA against S. aureus strains

Subinhibitory concentration of TA (99.0% purity, Sigma-Aldrich, Poznan, Poland) against *S*. *aureus* strains was determined by an agar dilution method according to the Clinical and Laboratory Standards Institute (CLSI) [16] guidelines with the modification described in detail in our previous study [14]. Briefly, several colonies were taken from cultured *S*. *aureus* strains on Columbia agar supplemented with 5% sheep blood, resuspended in sterile PBS (pH = 7.4), and washed three times. The bacterial suspension was introduced to a density of 0.5 on the McFarland scale and transferred using a multichannel pipette (32 spots; 10^4^ cfu/spot) to Mueller-Hinton agar (MHA, control), MHA supplemented with 1% (v/v) Tween 80 (Sigma-Aldrich, Poznan, Poland) and MHA supplemented with 1% (v/v) Tween 80 and TA at concentrations of 0.02–10%. Moreover, a sterility control was also considered (MHA). The media were prepared according to the procedure described in our previous study [14]. The plates were allowed to dry at room temperature and then incubated for 18 h at 37°C. After incubation, minimum inhibitory concentration (MIC_100_) was determined as the lowest TA concentration that inhibits the growth of *S*. *aureus* colonies. According to CLSI recommendations, a single colony or a faint haze caused by the inoculum was not considered. The subinhibitory concentration (MIC_50_) value (the minimum inhibitory concentration required to inhibit the growth of 50% of bacteria) was calculated proportionally as the MIC_100_ value. The study was conducted in duplicate.

At this stage, the following media were used for further studies: medium A–MHA (control); medium B–MHA medium supplemented with 1% (v/v) Tween 80; and medium C–MHA supplemented with 1% (v/v) Tween 80 and TA at MIC_50_.

### DNA isolation and detection of the chp gene

For isolation of total DNA from *S*. *aureus* strains, the commercial GeneMATRIX Bacterial & Yeast Genomic DNA Purification Kit (E3580, EURx, Gdansk, Poland) and lysostaphin (0.4 U/μl) (A&A Biotechnology, Gdansk, Poland) were used. All stages of DNA isolation were performed according to the manufacturer’s procedure. The obtained DNA was stored for further genetic analyses at -80°C.

The following specific pair of primers were used to amplify the *chp* gene by PCR: chp_1 (5’-GAA AAA GAA ATT AGC AAC AAC AG-3’), and chp_2 (5’-CAT AAG ATG ATT TAG ACT CTC C-3’) [3]. The following reaction mixture was added to each sample: bacterial DNA; 150–400 nM pair of specific primers (chp_1 and chp_2) (IBB-PAN, Gdansk, Poland); 5x concentrated GoTaq reaction buffer (Promega, Madison, WI, USA); 25 mmol/l MgCl_2_ (A&A Biotechnology, Gdansk, Poland), 10 mmol/l dNTPs mixture (A&A Biotechnology, Gdansk, Poland), 0.1 U/μl Hot-Start Taq DNA polymerase (A&A Biotechnology, Gdansk, Poland), and nuclease-free water. Amplification was performed using an Applied Biosystems Veriti 96 Well Thermal Cycler (Applied Biosystems, Norwalk, CT, USA) with the following protocol: 95°C for 4 min, then 30 cycles of 95°C for 15 s, 48°C for 50 s and 72°C for 60 s, followed by 72°C for 10 min [3]. *chp*-positive (*S*. *aureus* ATCC 25904), *chp*-negative (*S*. *aureus* ATCC 6538) strains, as well as a control reaction containing all reagents except the extracted DNA (no template control) were included in the study.

PCR products and 100-bp molecular size standard ladder (A&A Biotechnology, Gdansk, Poland) were added into 1.5% agarose gel (DNA Gdansk, Gdansk, Poland) in 1X Tris/borate/EDTA (TBE) buffer and ethidium bromide (Sigma-Aldrich, Poznan, Poland) as a staining agent. The electrophoresis was done at 100 V for 80 min, and the bands were visualized under UV light using a gel image system GelDoc-It2 Imager (Analytik Jena US LLC, Upland, CA, USA). The study was conducted in duplicate.

### RNA isolation and quantitative real-time PCR assay

One hundred mg of *S*. *aureus* biomass was collected from A-C media after 24 h of growth at 37°C in aerobic atmosphere. Biomass was homogenized in TRIzol (Invitrogen, Carlsbad, CA, USA) with 50 mg of 150–212 μm of glass beads (Sigma-Aldrich, Poznan, Poland). Three cycles of the beating of 2 min each, with 1 min incubation on ice within the cycles, were carried out in the Tissue Lyser LT (Qiagen, Hilden, Germany). RNA was extracted with chloroform (Sigma-Aldrich, Poznan, Poland). The upper aqueous phase was transferred to a new tube, and then RNA was precipitated with isopropanol (Chempur, Piekary Slaskie, Poland). After precipitation with isopropanol, RNA was washed twice with ethanol (Chempur, Piekary Slaskie, Poland). Next, RNA was resuspended in 50 μl of RNase-free water (Invitrogen, Waltham, MA, USA). RNA concentration was measured using a spectrophotometer (DeNovix DS-11 FX, Wilmington, DE, USA), and purity was verified by the ratio of absorbance at 260–280 nm. Moreover, RNA integrity was confirmed by agarose gel electrophoresis with ethidium bromide (Sigma-Aldrich, Hamburg, Germany) staining. RNA samples were treated with DNase I (Sigma-Aldrich, Hamburg, Germany) for 1 hour at 37°C. cDNA was synthesized using iScript™ cDNA Synthesis Kit (Bio-Rad, Hercules, CA, USA) according to the manufacturer’s instructions. The following primers were used in quantitative PCR (qPCR) study: 16S rRNA_1 (5’-GCT GCC CTT TGT ATT GTC-3’), 16S rRNA_2 (5’-AGA TGT TGG GTT AAG TCC C-3’) for *16S rRNA* [17], chp_1 (5’-GAA AAA GAA ATT AGC AAC AAC AG-3’), and chp_2 (5’-CAT AAG ATG ATT TAG ACT CTC C-3’) [3]. qPCR was conducted using CFX Connect™ Real-Time System (Bio-Rad, Hercules, CA, USA) and SsoFast EvaGreen Supermix (Bio-Rad, Hercules, CA, USA). The following reaction mixture was added to each sample: template cDNA, SsoFast EvaGreen Supermix, nuclease-free water, and 0.5 μM of each primer. Reaction mixtures were initially incubated for 30 s at 95°C, followed by 35 cycles at 95°C for 10 s and 15 s at 60°C. The specificity of PCR was evaluated by melt curve analysis in a temperature ranging from 95 to 58°C performed for each reaction. Electrophoresis was carried out in 1% agarose gel. Residual DNA contamination was checked in each RNA sample by running no reverse transcriptase controls. One reference gene was chosen (*16S rRNA*) from other candidate genes (*rpoB*, *gyrB*, *recA*, *rho*, *pta*, *rplD*, *tpo*) to identify stability under the influence of TA [18]. All samples were analyzed in triplicate. All experiments were repeated five times. *S*. *aureus* ATCC 25904 DNA was used as a positive control reaction. PCR efficiencies determined for all tested primer pairs were above 98%. Relative transcript levels were calculated according to Pfaffl (2001) [19]. Data were analyzed using Bio-Rad CFX Manager (Bio-Rad, Hercules, CA, USA) software.

### HL-60 cell culture

HL-60 cell line (human promyelocytic leukemia, HL60 ECACC 98070106, obtained in 2020) and all cell culture reagents were purchased from Sigma-Aldrich (Poznan, Poland). All sterile, disposable cell culture plasticware was purchased from VWR (Gdansk, Poland). After thawing, a recovery protocol [20] was conducted to ensure the complete removal of dimethyl sulfoxide (DMSO) used for cryopreservation, and a proliferating, undifferentiated HL-60 cell population was obtained. Briefly, a 1 ml aliquot of cells was gradually diluted with ~9 ml of RPMI-1640 media containing 20% FBS, 2 mmol/l L-glutamine, 100 U/ml penicillin, and 100 μg/ml streptomycin and then washed three times by centrifuging (10 min, 200 × g), aspirating the supernatant, and resuspending it in fresh media. Then, after 4 hours of culture at a density of 5 × 10^5^ cells/ml, the cells were washed once more and then cultured overnight. Over the next two days, the cells were washed daily (resuspended at 5 × 10^5^ cells/ml) and gradually weaned from 20% FBS to 10%. Then, over the next five days, the cells were also passaged by centrifugation and maintained at cell density between 1 × 10^5^ to 10 × 10^5^ cells/ml. Cell viability and density were monitored daily using trypan blue exclusion assay and hemocytometer counting. Before differentiation, the cell viability was ≥98% and doubling time ~24 h in RPMI-1640 growth media containing 10% FBS, 2 mmol/l L-glutamine, 100 U/ml penicillin, and 100 μg/ml streptomycin.

For differentiation of HL-60 cells into neutrophil-like cells (dHL-60), cells were centrifuged and resuspended at 1 × 10^5^ cells/ml in RPMI-1640 growth media (as noted above) with addition of 1.3% DMSO [21]. After three days of culture, cell viability and density were monitored daily, as described above, and dHL-60 cells were used for experiments on the 6th day of culture. Differentiation was manifested by a lack of proliferation, cell and nuclear morphology, and nitroblue tetrazolium (NBT) reduction assay [22]. 0.5 μg/ml of Hoechst 33342 dye (Cayman Chemical via VWR, Gdansk Poland) was added to the media to assess nuclear morphology. After 10 min, cells were imaged using Leica DMi8 (Leica Microsystems, Wetzlar, Germany) inverted fluorescence microscope with 40X (NA 0.6) focus and DAPI filter (Ex350/Em460). For the NBT assay, the cells were stimulated with 200 ng/ml phorbol 12-myristate 13-acetate (PMA) for 20 min at 37°C, and then a 1:1 volume of PBS containing 0.2% NBT was added, and >200 cells were counted with a hemocytometer. Undifferentiated HL-60 cells were used as controls in order to assess differentiation.

### Chemotaxis assay

The chemotactic response of dHL-60 cells to *S*. *aureus* strains was measured by QCM™ Chemotaxis 3-μm 96-well cell migration assay (ECM 515, Chemicon, Merck KGaA, Poznan, Poland) using Boyden chamber, according to the manufacturer’s protocol. Briefly, several colonies were taken from *S*. *aureus* strains grown on A-C media (for 18 h at 37°C), resuspended in Hank’s Balanced Salt Solution (HBSS, Biomed, Cracow, Poland), and washed three times to remove any particles or impurities. The bacterial suspension reached density of 0.5 on the McFarland scale. Duplicate samples of each bacterial suspension (10^4^ cfu/ml) and fMLP (100 μmol/l, control, Sigma-Aldrich, Poznan, Poland) were applied onto a 96-well feeder tray, whereas dHL-60 cells (5 × 10^4^ cells/100 μl) to the cell migration chamber plate. Cells were incubated for 2 h at 37°C in an incubator containing 5% CO_2_ and then fluorescently labeled using CyQuant GR dye according to the manufacturer’s instructions. The fluorescence intensity of each sample was measured on a microplate reader (Infinite 200 Pro, Tecan, Männedorf, Switzerland). A *chp*-positive (*S*. *aureus* ATCC 25904) and *chp*-negative (*S*. *aureus* ATCC 6538) strains were used as a control. The study was conducted in duplicate.

### Molecular docking

The TA molecule (ligand) was drawn manually by using Avogadro 1.1.1 [23] and optimized within the UFF force field [24] (5000 steps, conjugate gradient algorithm). The flexible, optimized ligand was docked onto the whole surface of the CHIPS protein. The structure deposited in the 1XEE.1 record of the PDB (www.rcsb.org) database was used for that purpose. Some fraction of the amino-acid sidechains was allowed to change the conformation; namely, the rotatable sidechains of those residues which maintain contact with the C5a receptor N-terminus in the C5a-CHIPS complex (according to the PDB:2K3U record). The AutoDock Vina software [25] was applied for docking simulations. The procedure of docking was carried out within the cuboid region which covered the CHIPS structure. The maximal number of poses generated during a single run was increased to 50, whereas the exhaustiveness parameter was increased to 16. Apart from that, all default procedures and algorithms implemented in AutoDock Vina were kept.

### Molecular dynamics simulation

Molecular dynamics (MD) simulations concerned a single CHIPS protein (of structure relying on the PDB:1XEE.1 record), interacting with 10 TA molecules and immersed in explicit water with addition of Na^+^ and Cl^-^ ions neutralizing the total charge and maintaining the ionic strength of 0.15 M. All MD simulations were carried out with the GROMACS 2016.1 package [26]. The GROMOS 54a7 force field [27] was used and the parameters generated by Automated Topology Builder [28] were adopted to describe the TA molecules. The CHIPS molecule was placed in cubic simulation box of initial dimensions of 6.6 × 6.6 × 6.6 nm and surrounded by ~9000 explicit water molecules (simple point charge model [29]) and ca. 30 Na^+^ and Cl^-^ ions. The MD simulations were carried out under periodic boundary conditions and in the isothermal-isobaric ensemble. The temperature was maintained close to its reference value (37°C) by applying the V-rescale thermostat [30], whereas for the constant pressure (1 bar, isotropic coordinate scaling), the Parrinello-Rahman barostat [31] was used with a relaxation time of 4 ps. The equations of motion were integrated with a time step of 2 fs using the leap-frog scheme [32]. The solute bond lengths were constrained by application of the LINCS procedure with a relative geometric tolerance of 10^−4^ [33]. The full rigidity of the water molecules was enforced by application of the SETTLE procedure [34]. The translational center-of-mass motion was removed every timestep separately for the solute and the solvent. The non-bonded interactions were calculated using a single cutoff distance set to 1.4 nm and a Verlet list scheme. The reaction-field correction was applied to account for the mean effect of electrostatic interactions beyond the long-range cut off distance, using a relative dielectric permittivity of 61 as appropriate for the SPC water model [35]. All the systems were preoptimized by a series of constant-pressure, constrained simulations (lasting 2–10 ns). After these steps, production simulation was carried out for a duration of 500 ns and the trajectory was saved every 10 ps. The MD trajectory was assessed with the GROMACS *mdmat* tool, analyzing the matrix of contacts between different components of the molecular system.

### Statistics

The statistical significance of chemotaxis results was assessed using a one-way ANOVA test. The Mann–Whitney test was used to analyze *chp* gene expression between TA-treated (cultivated on medium C) and non-treated (cultivated on medium A) strains. The following values: *P<0.05, **P<0.01, ***P<0.001 were considered statistically significant. Statistical analyses were performed using GraphPad Prism 5.02 (GraphPad Software Inc., San Diego, CA, USA) and Statistica version 12 (StatSoft, Inc, Palo Alto, CA, USA) software.

## Results

### Evaluation of the antibacterial activity of TA against S. aureus strains

The applied agar dilution method confirmed that TA demonstrates antibacterial activity against both *S*. *aureus* reference strains and isolates. Obtained MIC_100_ and MIC_50_ values of TA ranged from 2.5 ± 0.0 to 10.0 ± 0.0% and 1.3 ± 0.0 to 5.0 ± 0.0%, respectively (see S1 Fig in S1 File). It was also noted that the supplementation of MHA by Tween 80 (1%, v/v) had no impact on growth inhibition of *S*. *aureus* strains, which was also observed in our previous study [14].

### PCR and PFGE results

The PCR assay showed that 50% of isolates were *chp*-positive (see S2 Fig in S1 File). Moreover, genetic relatedness analysis of the tested isolates using the PFGE method as well as the FPQuest program (similarity coefficient [SA_B_] value = 53.5%) identified three PFGE genotypes (A, B, and C) and one unique pattern (U) (see S3 Fig in S1 File). It was also observed that *S*. *aureus* ATCC 25904 and *S*. *aureus* ATCC 6538 strains belonged to A and C genotypes, respectively.

### qPCR results

**Fig 1:**
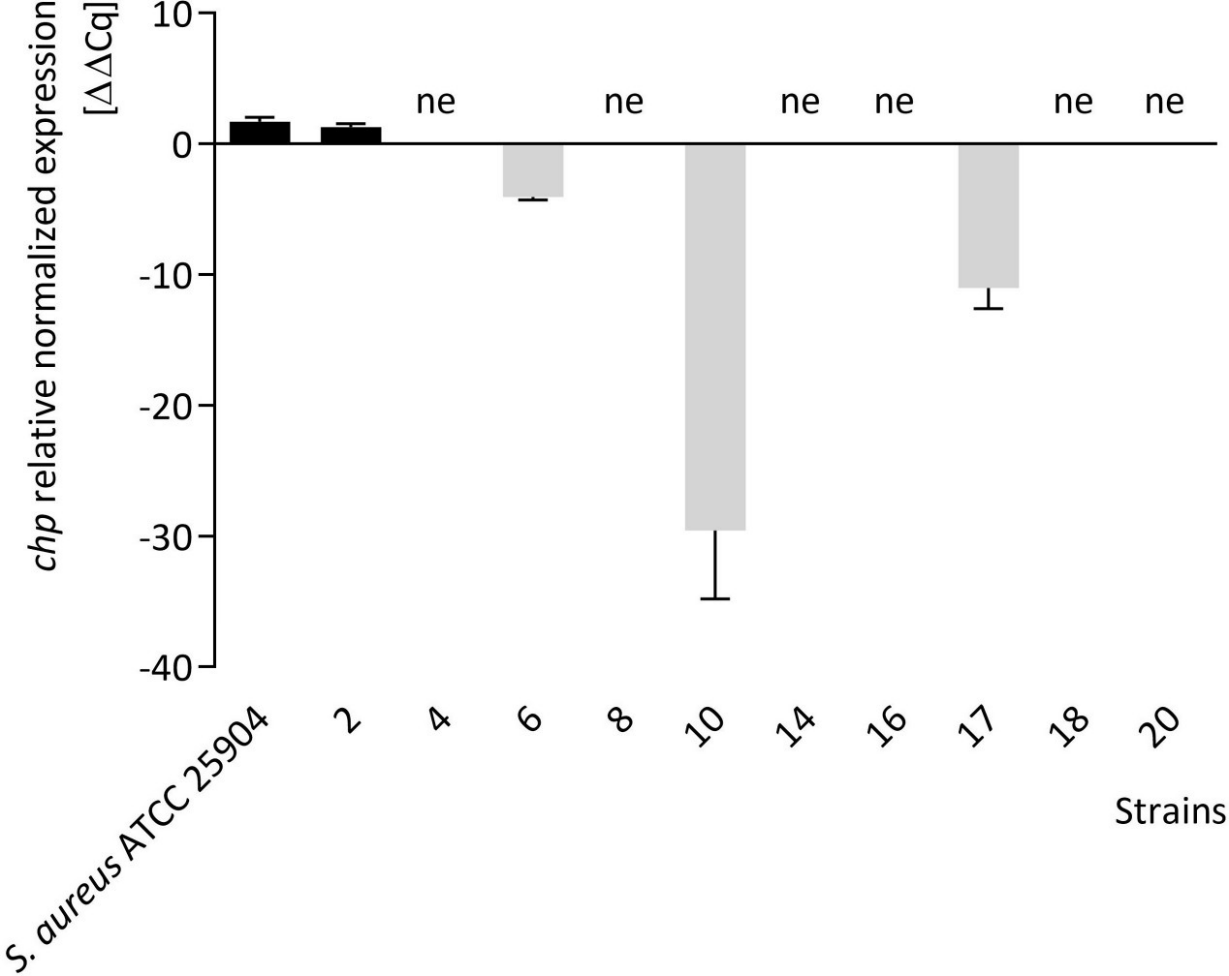
Relative expression level of the *chp* gene in *Staphylococcus aureus* strains cultured on Mueller-Hinton agar (MHA) supplemented with 1% (v/v) Tween 80 and *trans*-anethole (TA) at the subinhibitory concentration. The relative gene expression levels presented in the figure are a result of comparing the expression levels on the MHA (control–medium A) with MHA supplemented with TA (medium C). Values >0 indicates upregulation (black bars); values <0 indicates down-regulation (gray bars); ne–no expression. Data are shown as the mean ± standard deviation.

The *chp* gene expression assay was performed using *chp*-positive strains cultivated on A-C media. Based on the analyses, six *S*. *aureus* isolates showed no expression of the *chp* gene after incubation on a medium supplemented with MIC_50_ of TA (medium C) (Fig 1 and S4 Fig in S1 File). The reduction in mRNA levels was thirty-fold (*P*<0.01), eleven-fold (*P*<0.05), and four-fold (*P*<0.05), respectively, for strains no. 10, 17, and 6. Only *S*. *aureus* ATCC 25904 strain and strain no. 2 showed a 1.7-fold (*P*<0.05) and 1.3-fold (*P*<0.05) increase in *chp* transcription, respectively. As shown in S4A Fig of S1 File, a statistically significant difference (*P*<0.001) was observed in the reduction of *chp* gene expression in strains cultured on medium C compared to the control medium (without chemicals, A) and medium supplemented with Tween 80 (B). Moreover, no difference in expression was observed between medium A and B, indicating that the addition of Tween 80 (1%, v/v) did not affect the expression of the *chp* gene. The detailed data about relative normalized expression levels of the *chp* gene obtained in this study are listed in S2 Table of S1 File.

### HL-60 cell differentiation

HL-60 cell differentiation to neutrophil-like cells after six days of 1.3% DMSO treatment was confirmed based on loss of proliferative ability, changes in nuclear morphology, and the NBT assay for respiratory burst (see S5 Fig in S1 File). In terms of proliferation, after three days of exposure to 1.3% DMSO, dHL-60 cell density typically increased from 1 × 10^5^ cells/ml to ~6 × 10^5^ cells/ml, while over the same period, the density of control HL-60 cells increased to ~10 × 10^5^ cells/ml. Over the subsequent three days in culture, the density of dHL-60 remained stable with a terminal value of ~6.4 × 10^5^ cells/ml, confirming the loss of proliferative phenotype and indicating that cells had undergone maturation. Cell viability of dHL-60 cells was slightly lower than that of control HL-60 but remained high at 94–95%. Granulocytic differentiation was further confirmed by Hoechst 33342 staining, which revealed a marked change in nuclear morphology from round to polymorphic. Finally, the NBT assay confirmed that dHL-60 was capable of respiratory burst in response to PMA stimulation, with >90% of dHL-60 cells exhibiting dark blue-black granules.

### Chemotaxis results

**Fig 2:**
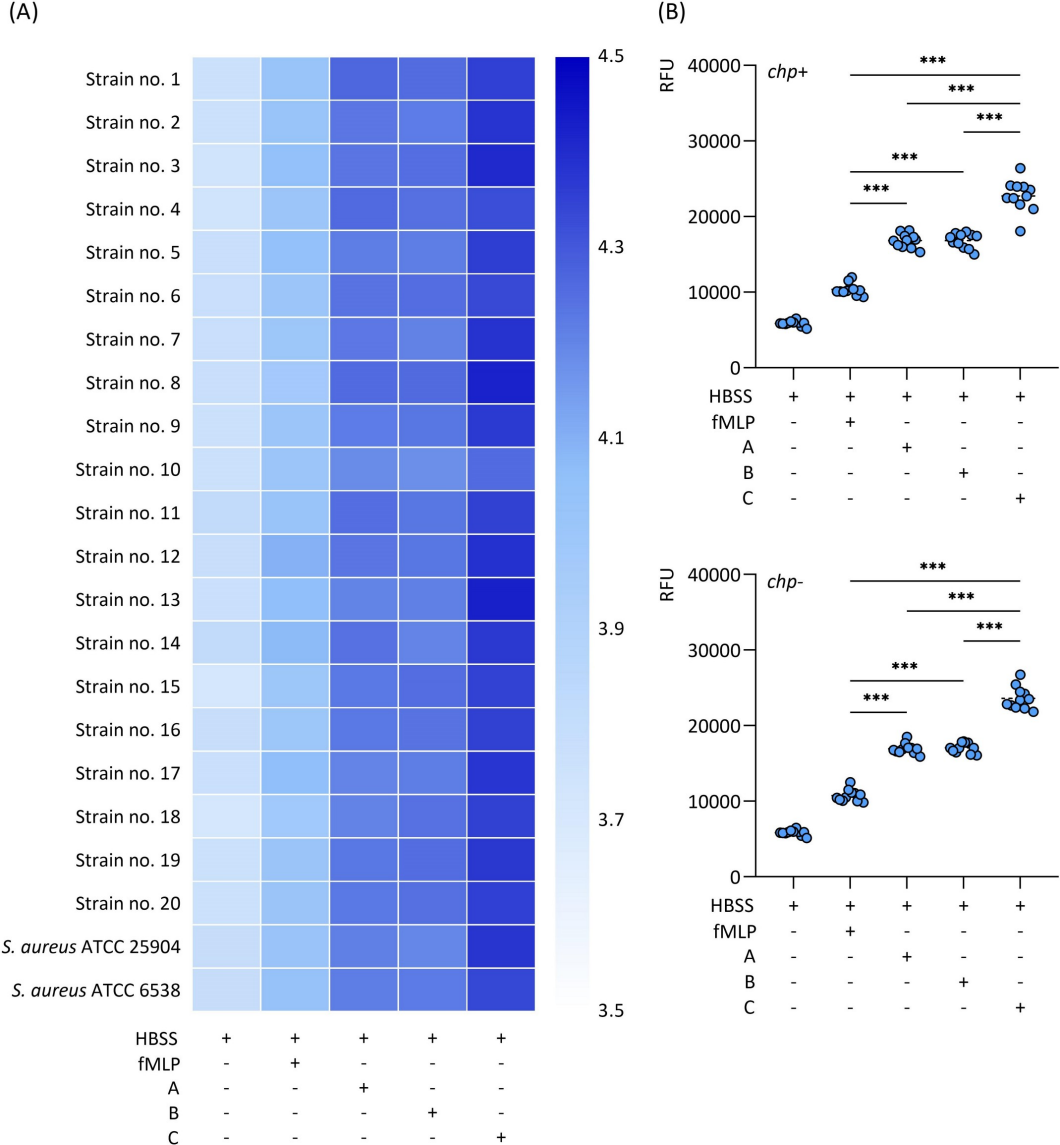
The differentiated HL-60 neutrophil-like cells chemotactic response to *trans-*anethole-treated *Staphylococcus aureus* strains. The heatmap (A) represents a log-mean relative fluorescence units (RFU) (+4.5 dark blue to 3.5 light blue). Scatter plots (B) demonstrating the distribution of mean RFU level for *chp*-positive (*chp*+) and *chp*-negative (*chp*-) *S*. *aureus* strains. A–Mueller-Hinton agar non-supplemented (MHA, control), B–MHA supplemented with 1% (v/v) Tween 80, and C–MHA supplemented with 1% (v/v) Tween 80 and subinhibitory concentration of *trans*-anethole. Control–N-formyl-L-methionyl-L-leucyl-phenylalanine (fMLP, 100 μmol/l), HBSS–Hank’s Balanced Salt Solution. *** *P*<0.001.

Using a Boyden chamber assay, it was revealed that migration of dHL-60 cells was enhanced by *S*. *aureus* cells (cultured on media A-C) than the control sample (fMLP) with significant *P*-values (<0.001) (Fig 2A). Interestingly, it has been also noted that the TA-treated bacteria caused statistically significant (*P*<0.001) increased chemotactic activity of dHL-60 cells compared to non-treated bacteria (cultured on media A and B). It is worth emphasizing that this correlation was similar for both *chp*-positive or *chp*-negative TA-treated *S*. *aureus* strains (Fig 2B). Overall, similar relative fluorescence units level was noticed between dHL-60 cell chemotactic response to *chp*-positive and *chp*-negative *S*. *aureus* strains.

### Molecular docking and MD simulation results

**Fig 3:**
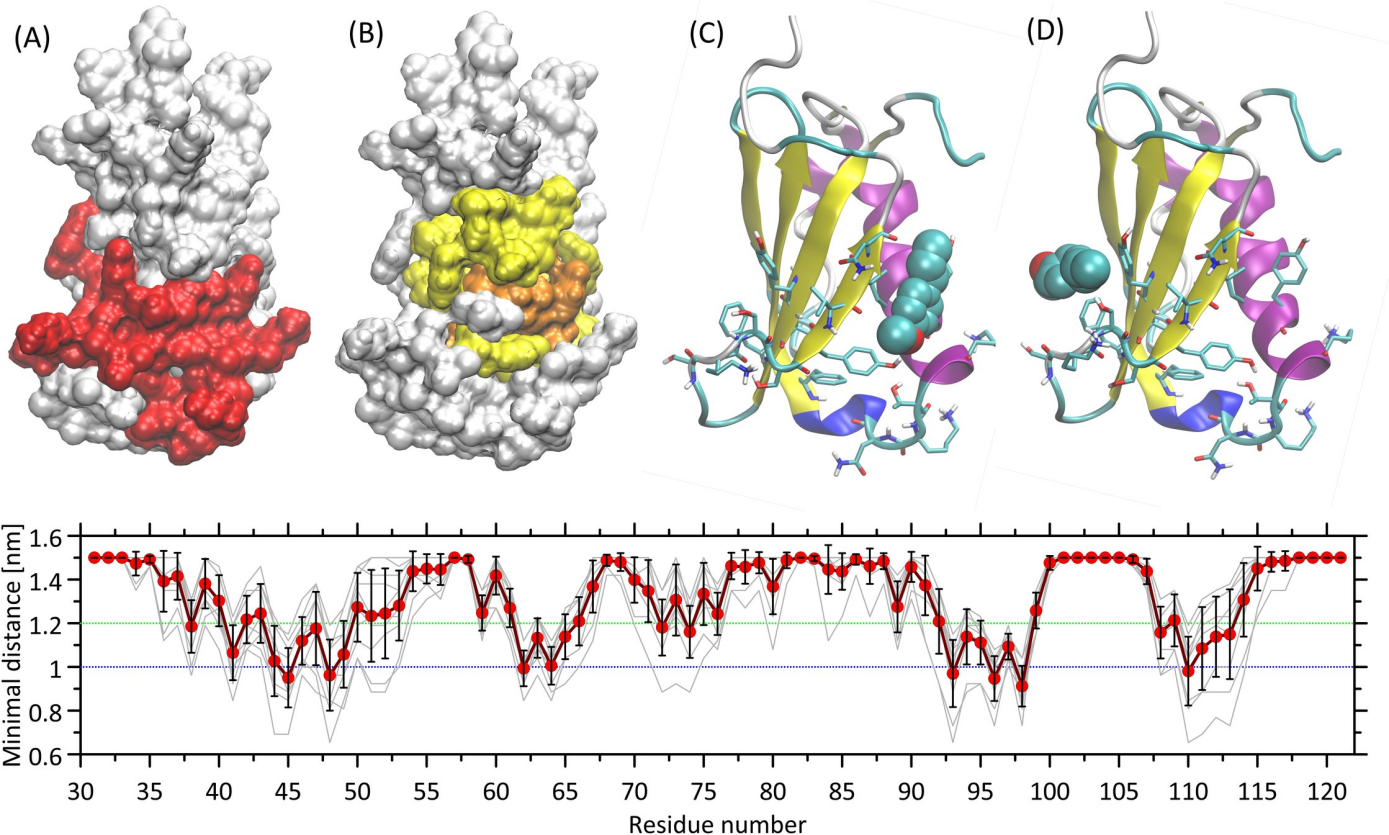
The graphical illustration of molecular modeling results. (A) The surface representation of the CHIPS protein (PDB:2KU3) with amino-acid residues maintaining contact with the C5a receptor N-terminus marked in red. The contact is defined as the minimal distance between any atom pair smaller than 0.25 nm. (B) The MD simulation results depicted as the amino-acid residues that are most prone to exhibit contact with the TA molecules during unbiased MD simulations; the residues displaying average, minimal distance below 1.2 nm and 1 nm are shown in yellow and orange, respectively. (C, D) The two most favorable locations of TA interacting with CHIPS that were found during docking procedure. The rotatable sidechains of amino-acid residues belonging to the C5a/CHIPS interface are marked as sticks, whereas the TA molecule is represented as van der Waals spheres. (*bottom panel*) Quantitative results showing the average, minimal distance between TA molecules and all amino-acid residues of CHIPS. The thin gray lines represent individual contributions from each of the 10 TA molecules present in the system, whereas the thick, red line shows the average value. The vertical bars represent the standard deviations. The dotted green and blue lines correspond to cutoffs defining the residues shown in the panel (B).

Docking calculations showed that the TA molecule is capable of binding favorably to the CHIPS surface in several different regions. However, the most favorable energies (varying within the -5.1 –-4.9 kcal/mol range) are associated with binding to the amino-acid residues that create the interface of the C5a/CHIPS contact. More precisely, binding of TA with contribution of non-polar (Ile, Leu, Val) and aromatic (Tyr, Phe) residues occurs, supported by hydrogen bonding where the role of donors is played by hydroxyl-containing amino-acid residues (Tyr, Ser and Thr). The two most favorable binding sites are located at the limiting edges of the C5a binding interface, in the vicinity of either Tyr48/Ile110 (Fig 3C) or Tyr97/Phe99 (Fig 3D). Several distinct orientations of TA, corresponding to approximately the same binding sites were detected which suggests high mobility of the bound TA molecule and reversible binding. The binding mechanism is controlled by the CH-π and π-π interactions (involving Tyr and Phe), non-polar interactions which are favorable due to reducing the area of solvent-accessible non-polar surfaces (Ile, Leu and Val) and above-mentioned hydrogen bonding.

The MD simulation results confirm the observations derived from docking. TA is capable of binding to the CHIPS surface and, although no single, well-defined binding site can be defined, certain regions of protein are strongly preferred for binding whereas other are unfavored. Fig 3A and 3B show a comparison of binding regions for TA, predicted by MD simulations with residues involved in binding the C5a receptor, as indicated by independent, structural data. In contrast to docking results only a single but broad region exhibiting the largest contribution to the TA binding was identified. This region centers around Tyr97 and the most intensive contacts include neighboring residues: Thr96, Phe98, Met62 and Ile110. Apart from that, some other residues, also close to the C5a/CHIPS interface, are involved, such as Tyr48, Leu45 and Tyr58. A relatively large area defined by these residues covers the both most favorable binding sites identified during docking. Let us also note that binding of TA in the regions distant from the C5a/CHIPS interface is essentially absent.

Both the similar binding profiles exhibited by different TA molecules (gray lines in Fig 3 [bottom panel]) and the fact that minimal average distance is always well above the distances expected on the basis of tight van der Waals contacts, indicate that the process of TA binding is highly reversible and the binding/unbinding events occurred multiple times throughout the MD simulations. Apart from treating this observation as an evidence of equilibration and convergent results, it can also be stated that, under standard conditions, the TA affinity for CHIPS is of rather moderate magnitude and the degree of saturation of the binding site will be highly dependent on the TA concentration around CHIPS.

## Discussion

The body first line of defense is activated during the inflammatory response triggered by extracellular bacteria. Many chemotactic factors (e.g. fMLP, C5a, or IL-8) act on phagocytes in the vascular bed, finally leading them to the target [36]. The consequence of phagocyte influx to the site of the inflammatory response is phagocytosis, being a major process of the innate immune response carried out in the phagolysosome, which involves antimicrobial peptides and reactive oxygen species produced during the oxidative burst. The final step in phagocytosis is programmed phagocyte death [37].

Over the past several years, it has been noted that *S*. *aureus* has begun to evolve and resist an increasingly innate immune response, including chemotaxis, initiating the process of phagocytosis. To date, it has been demonstrated that this process can be impaired by the following virulence factors produced by this pathogen: FPR-like 1 inhibitory proteins [38], staphopain A [39], extracellular adherence protein [40], staphylococcal-like proteins (e.g. Ssl7) [41], and CHIPS [10].

The present study focused, among other things, on chemotactic response of dHL-60 cells to *S*. *aureus* strains pre-cultured on the medium containing a subinhibitory concentration of TA. So, the first step was to investigate the antimicrobial properties of TA against analyzed strains. This study revealed that all strains were susceptible to TA, which was also confirmed in our previous studies [42]. According to the MIC values of TA, subinhibitory concentrations were determined and used for further steps in the study. Moreover, it has been shown that DNA fingerprinting by PFGE assigned the staphylococcal strains to 4 distinct clusters.

Available literature lacks studies on pretreatment of bacteria with natural compounds and their subsequent effect on chemotaxis. However, there are many reports on direct effects of these compounds on this process. To date, increased neutrophil migration towards jungle honey [43] or polysaccharides purified from *Ganoderma lucidum* [44] has been demonstrated. Inhibition of neutrophil chemotactic responses has been observed in the presence of, viridiflorol [45], sabinene, isobornyl acetate, β-pinene, γ-terpinene, geranyl acetone [46], or methanol extract of *Garcinia staudtii* twigs [47]. Interestingly, it was also noted that eugenol and even TA showed an inhibitory effect on neutrophil migration [48].

Studies performed by Schmeling et al. [49], Riber et al. [50], or von Aulock et al. [51] found that whole *S*. *aureus* cells, their cell wall, isolated peptidoglycan, or lipoteichoic acids influenced the increased chemotactic activity. Based on our former observations, it was found that the TA-treated *S*. *aureus* ATCC 25904 strain was characterized by changes, e.g. in the cell wall structure compared to a non-treated strain [14]. What is more, an increased IL-8 level in a human whole-blood model infected with this strain was also revealed. We assumed that those changes observed in the structure of the cell wall of TA-treated *S*. *aureus* (among others, increased content of peptidoglycan which is considered the main pathogen-associated molecular pattern of *S*. *aureus*) could stimulate the immune system more to fight bacteria. Considering the above results, it can be probably assumed that also, in this case, changes in *S*. *aureus* cell surface structure observed in our previous studies [14] may play a pivotal role in the increased chemotactic potential. Nevertheless, further analyses are required to understand this mechanism better.

To the best of our knowledge, this is also the first study showing the effect of TA on *chp* gene expression. Literature data indicate that the *chp* gene is characterized by early expression and translation, resulting in rapid production of CHIPS in the early phase of the infection [10]. It has also been shown that its level is increased in early stages of biofilm formation [52]. A model proposed by Kwiecinski et al. [53] showed that *chp* gene expression is dependent on ArlS/R and MgrA global regulators. Furthermore, Chroboczek et al. [54] report that the presence of the *chp* gene is significantly associated with ST398 (CC398 MSSA), human isolates capable of colonizing nares, as well as causing a broad spectrum of infections.

In the current study, it has been demonstrated that TA reduced or inhibited *chp* gene expression in most of the analyzed isolates. Interestingly, it has been also observed that TA increased *chp* gene expression in two strains (*S*. *aureus* ATCC 25904 and strain no. 2), which, however, did not correlate with the chemotactic response of dHL-60 cells. Overall, it has been proven that regardless of whether the expression of the *chp* gene was completely inhibited or significantly reduced (9 strains), increased (2 strains) or absent (9 strains), the chemotaxis of dHL-60 towards TA-treated bacteria increased. On this basis, we can assume that *chp* is probably not the key factor in the tested strains that is responsible for the increased chemotaxis under TA influence. Nevertheless, further analyses are required to understand this mechanism better.

A lack of CHIPS ELISA tests on the market makes it very difficult to study the effect on expression at the level of translation. Since an increase in *chp* gene expression in two staphylococcal strains after TA treatment was noticed in the current study which can be probably correlated with later production of CHIPS [10], molecular docking and MD simulation studies of interactions of TA with CHIPS were performed. Both docking calculations and MD simulations provide a consistent picture of the CHIPS-TA interactions. TA is preferentially bound in the region of the CHIPS groove responsible for interactions with the N-terminus of the C5a receptor and can interfere with any processes exploiting this binding site. The dynamic, reversible character of binding suggests that the observed consequences of prospective alterations will be strongly dependent on the TA concentration.

This study has potential limitations. For instance, due to the fact that in the present study, the chemotaxis assay was performed using TA-treated bacteria, at this stage it is extremely difficult to establish the correlation between the *chp* gene expression under TA influence and simultaneous chemotactic response of dHL-60 cells in HBSS after 2 hour incubation. Nevertheless, so as to provide stronger evidence, it is advisable to confirm our observations at different time intervals and using a larger number of strains (especially with different antibiotic resistance phenotypes). The correlation of the above results with CHIPS protein production is also relevant here.

## Conclusions

In conclusion, it could be said that dHL-60 cells exhibited a higher chemotactic response to TA-treated *S*. *aureus* strains in comparison to non-treated bacteria. Interestingly, pretreatment of *S*. *aureus* with TA resulted in reduction/inhibition of *chp* expression in most isolates. However, the enhanced chemotactic activity of dHL-60 cells to TA-treated bacteria was independent of the *chp* expression level. Moreover, both molecular docking and MD simulations studies confirmed that TA is preferentially bound in the C5a/CHIPS interface interaction region and can interfere with any processes exploiting this binding cavity.

It can be assumed that also, in this case, changes in *S*. *aureus* cell surface structure observed in our previous studies [14] may probably play a pivotal role in the increased chemotactic potential. The presence of other virulence factors, undergoing TA-mediated expression, cannot be ruled out here, either. Hence, further analyses are required to understand this mechanism better.

## Supporting information

S1 File Contains all the supporting tables and figures. (DOCX)
