# Supplementary material for: Enhancement of neutrophil chemotaxis by trans-anethole-treated Staphylococcus aureus strains
Source: PLoS One. 2023 Apr 7;18(4):e0284042. doi: 10.1371/journal.pone.0284042 (PMC10081771; doi:10.1371/journal.pone.0284042)
Supplement: S1 File — (DOCX) [file pone.0284042.s001.docx]

**Supporting Information**

**Enhancement of neutrophil chemotaxis by *trans*-anethole-treated *Staphylococcus aureus* strains**

Paweł Kwiatkowski^1,*^, Aleksandra Tabiś^2^, Peter Sobolewski^3^, Wojciech Płaziński^4,5^, Agata Pruss^6^, Monika Sienkiewicz^7^, Barbara Dołęgowska^6^, Iwona Wojciechowska-Koszko^1^

^1^Department of Diagnostic Immunology, Pomeranian Medical University in Szczecin, Poland

^2^Department of Food Hygiene and Consumer Health Protection, Wroclaw University of Environmental and Life Sciences, Wroclaw, Poland

^3^Department of Polymer and Biomaterials Science, Faculty of Chemical Technology and Engineering, West Pomeranian University of Technology, Szczecin, Poland

^4^Jerzy Haber Institute of Catalysis and Surface Chemistry, Polish Academy of Sciences, Krakow, Poland

^5^Department of Biopharmacy, Medical University of Lublin, Poland

^6^Department of Laboratory Medicine, Pomeranian Medical University in Szczecin, Poland

^7^Department of Pharmaceutical Microbiology and Microbiological Diagnostic, Medical University of Lodz, Poland

***Corresponding author**:

Paweł Kwiatkowski, PhD

E-mail address: pawel.kwiatkowski@pum.edu.pl

**
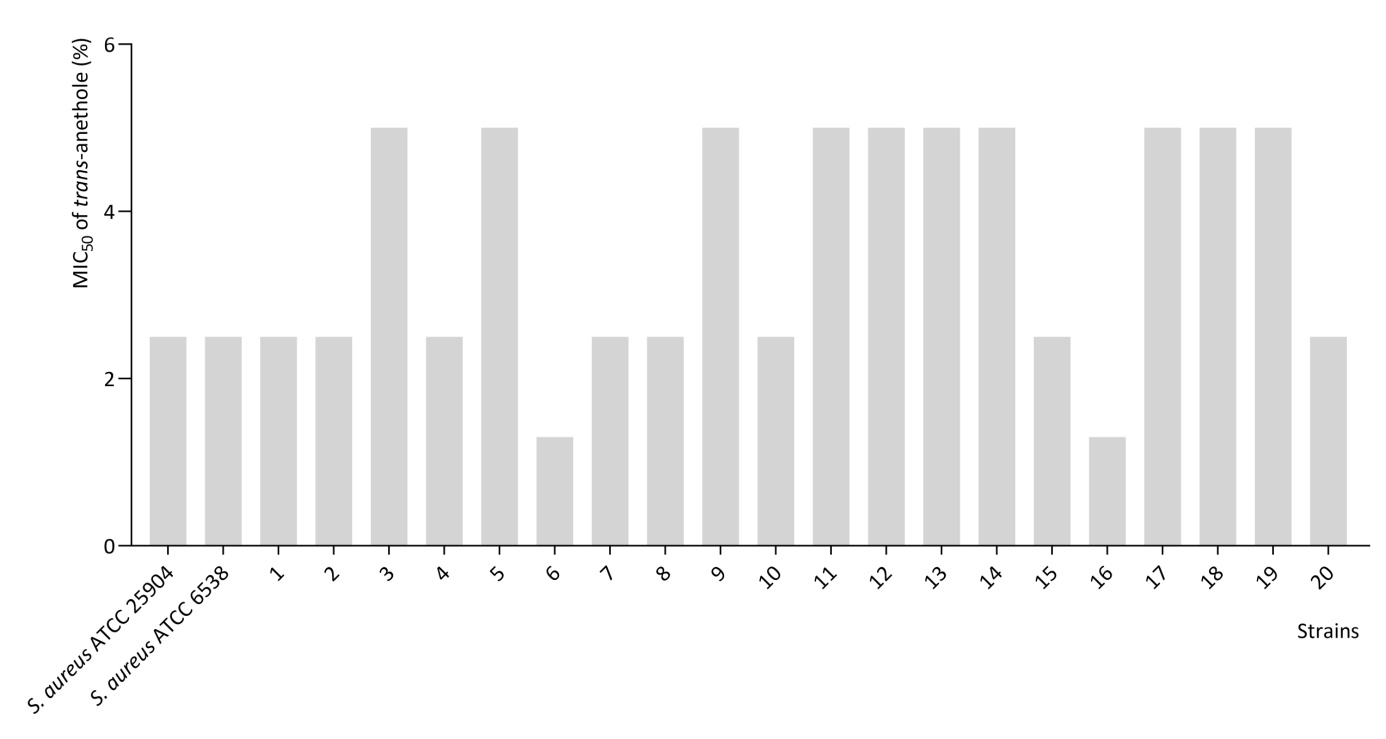
**

**S1 Fig.** ***trans*-Anethole susceptibility testing of *Staphylococcus aureus* strains.** MIC_50_ – minimum inhibitory concentration required to inhibit the growth of 50% bacteria.

**
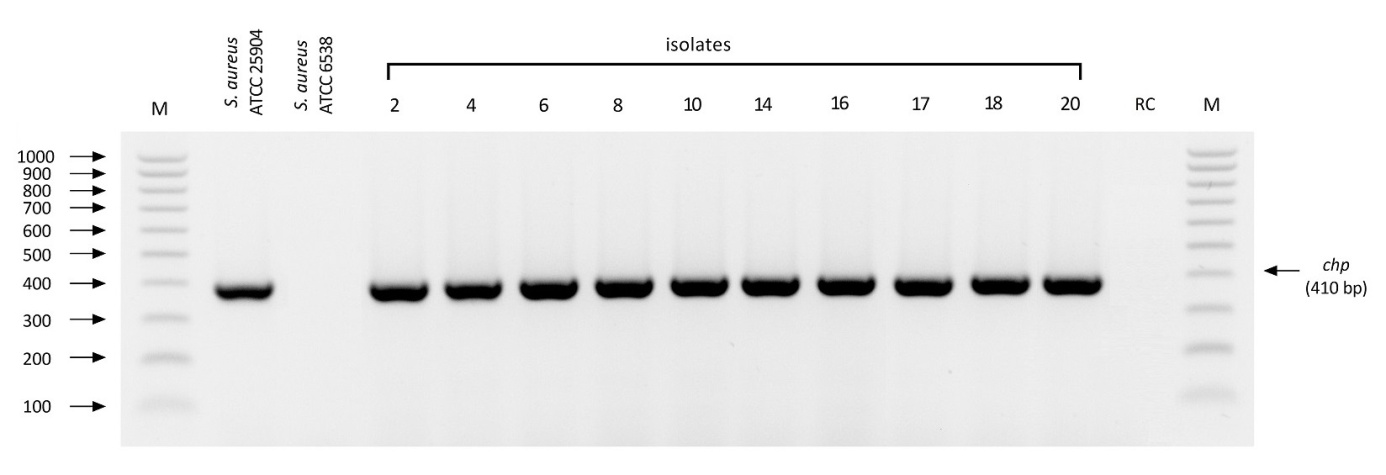
**

**S2 Fig.** **Electrophoresis in 1.5% agarose gel PCR products obtained by using specific primers for *chp* gene.** *Staphylococcus aureus* ATCC 25904 – *chp*-positive strain (positive control), *S. aureus* ATCC 6538 – *chp*-negative strain (negative control), M – molecular weight, BP – base pair, RC – control reaction containing all reagents except the extracted DNA (no template control).

**
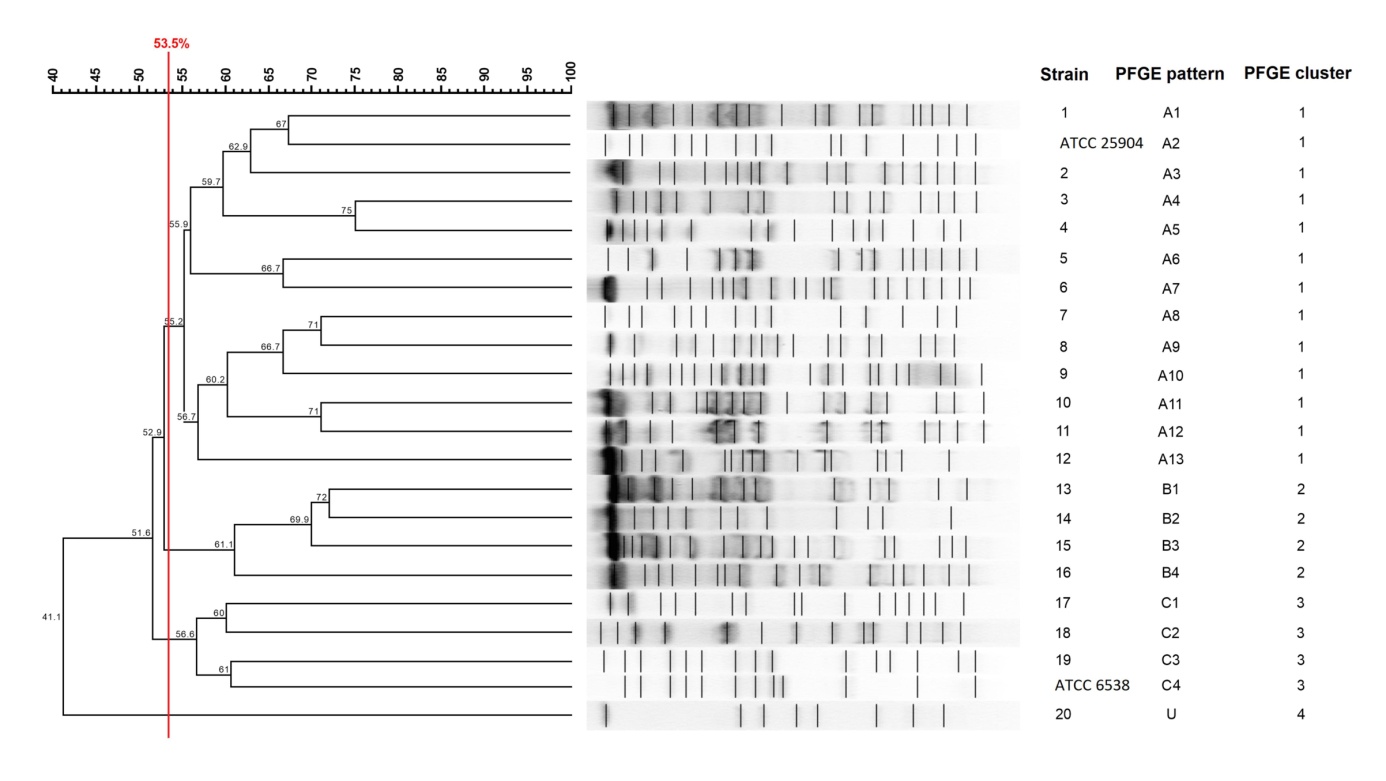
**

**S3 Fig. Pulsed-filed gel electrophoresis (PFGE) of analyzed strains.** U – unique strain.

**
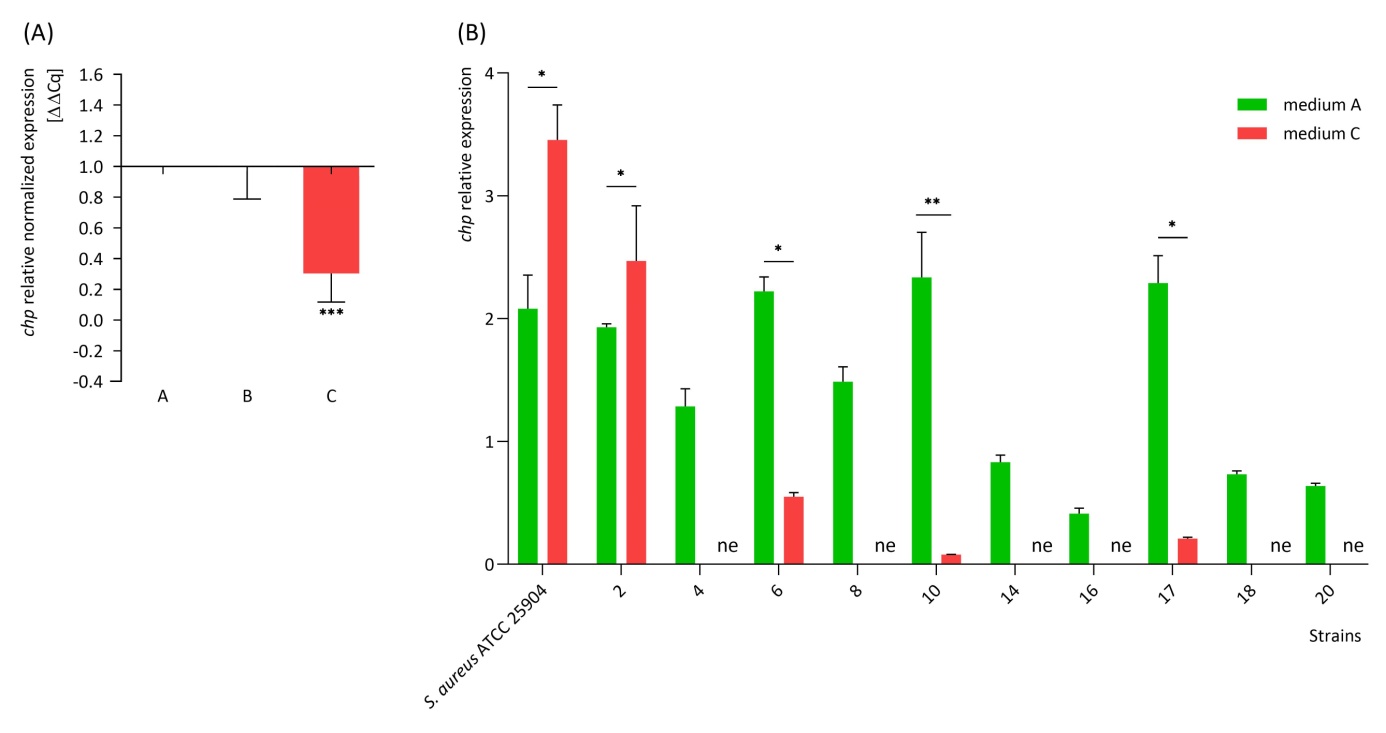
**

**S4 Fig.** **Normalized (A) and relative (B) expression level of *chp* gene in *Staphylococcus aureus* strains cultured on Mueller-Hinton agar: non-supplemented (control – medium A); supplemented with 1% (v/v) Tween 80 (medium B); supplemented with 1% (v/v) Tween 80 and *trans*-anethole at the subinhibitory concentration (medium C).** ne – no expression. Data are shown as the mean ± standard deviation. **P*<0.05, ***P*<0.01, ****P*<0.001.

**
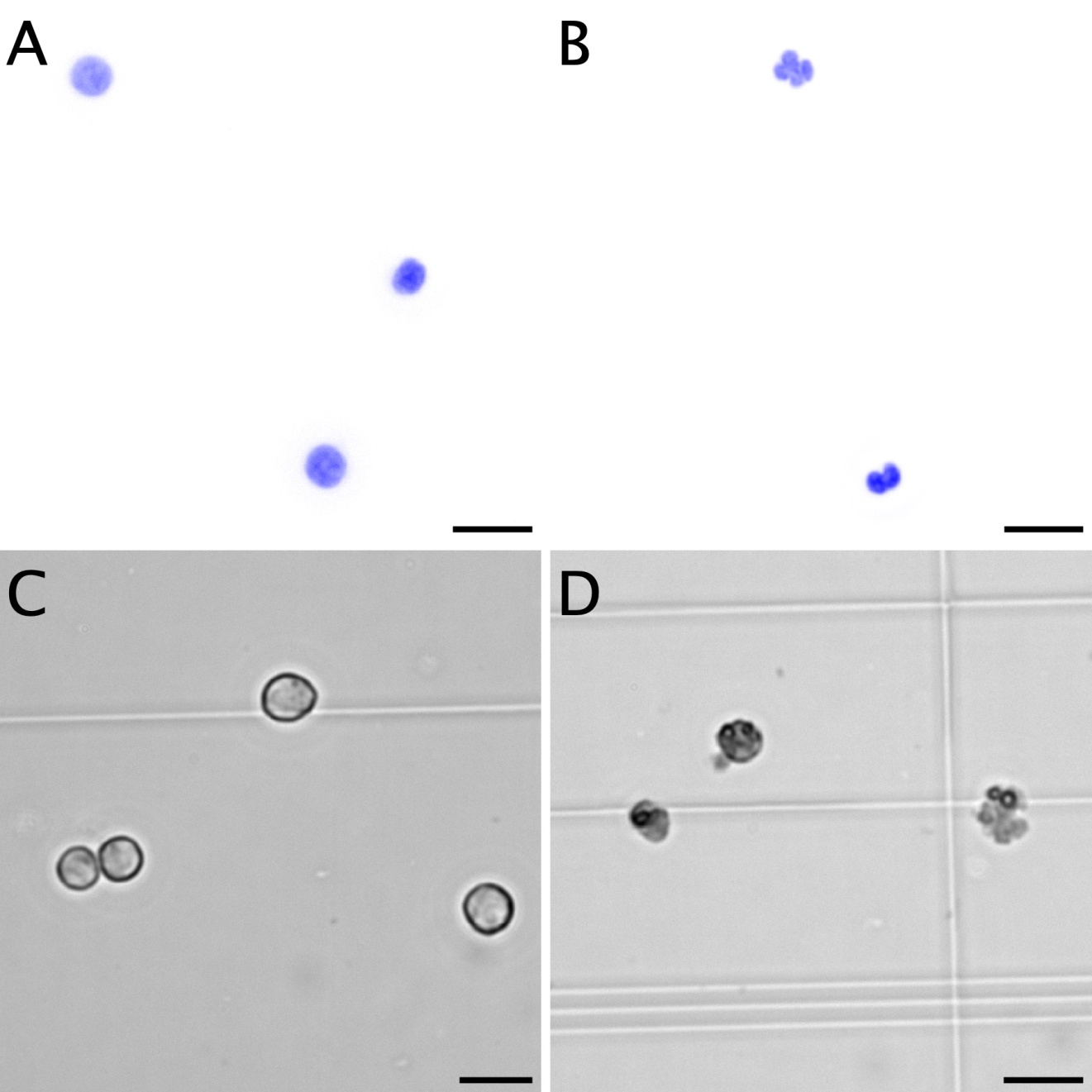
**

**S5 Fig.** **Representative micrographs of control (A, C) and differentiated (B, D) HL-60 neutrophil-like cells.** Top row (A, B): nuclear staining with Hoechst 33342. Bottom row (C, D): NBT staining after phorbol 12-myristate 13-acetate stimulation. Scale bars represent 20 µm.

**S1 Table. Minimum inhibitory concentration (MIC) of oxacillin and cefoxitin against *Staphylococcus aureus* strains.**

| **Strain** | **MIC of oxacillin**  **[mg/l]** | **MIC of cefoxitin**  **[mg/l]** |
| --- | --- | --- |
| *Staphylococcus aureus* ATCC 25904 | 0.125 | 0.38 |
| *Staphylococcus aureus* ATCC 6538 | 0.25 | 0.38 |
| 1 | 0.5 | 1.0 |
| 2 | 0.25 | 0.38 |
| 3 | 0.063 | 0.125 |
| 4 | 0.125 | 0.38 |
| 5 | 0.094 | 0.38 |
| 6 | 0.5 | 1.5 |
| 7 | 0.25 | 0.25 |
| 8 | 0.25 | 0.5 |
| 9 | 0.094 | 0.125 |
| 10 | 0.5 | 1.0 |
| 11 | 0.25 | 0.25 |
| 12 | 0.125 | 0.25 |
| 13 | 0.25 | 0.25 |
| 14 | 0.094 | 0.25 |
| 15 | 0.25 | 0.38 |
| 16 | 0.125 | 0.25 |
| 17 | 0.125 | 0.25 |
| 18 | 0.094 | 0.38 |
| 19 | 0.25 | 0.5 |
| 20 | 0.5 | 0.75 |

MIC of oxacillin ≤2 mg/l or MIC of cefoxitin ≤4 mg/l is determined as methicillin-susceptible *S. aureus* (MSSA); MIC of oxacillin >4 mg/l or MIC of cefoxitin ≥8 mg/l is determined as methicillin-resistant *S. aureus* (MSSA) [1].

**References**

1. CLSI, Clinical and Laboratory Standards Institute. Performance Standards for Antimicrobial Susceptibility Testing; Twenty-Eighth Informational Supplement. Document M100-S28. CLSI (2018), Wayne, PA, USA.

**S2 Table. Relative normalized expression level of *chp* gene obtained in this study.**

| **Strain** | **Media** | **∆∆Cq ± SEM** |
| --- | --- | --- |
| *Staphylococcus aureus* ATCC 25904 | A | 2.08 ± 0.27 |
|  | B | 2.15 ± 0.23 |
|  | C | 3.46 ± 0.28 |
| 2 | A | 1.93 ± 0.03 |
|  | B | 1.91 ± 0.01 |
|  | C | 2.47 ± 0.45 |
| 4 | A | 1.29 ± 0.14 |
|  | B | 1.55 ± 0.08 |
|  | C | ne |
| 6 | A | 2.22 ± 0.12 |
|  | B | 2.09 ± 0.10 |
|  | C | 0.55 ± 0.03 |
| 8 | A | 1.49 ± 0.12 |
|  | B | 1.32 ± 0.08 |
|  | C | ne |
| 10 | A | 2.34 ± 0.37 |
|  | B | 2.56 ± 0.44 |
|  | C | 0.08 ± 0.002 |
| 14 | A | 0.83 ± 0.06 |
|  | B | 0.59 ± 0.10 |
|  | C | ne |
| 16 | A | 0.41 ± 0.05 |
|  | B | 0.48 ± 0.01 |
|  | C | ne |
| 17 | A | 2.29 ± 0.22 |
|  | B | 2.38 ± 0.19 |
|  | C | 0.21 ± 0.01 |
| 18 | A | 0.73 ± 0.03 |
|  | B | 0.59 ± 0.07 |
|  | C | ne |
| 20 | A | 0.64 ± 0.02 |
|  | B | 0.48 ± 0.05 |
|  | C | ne |

SEM – standard error of measurement; A – Mueller-Hinton agar (MHA) non-supplemented (control); B – MHA supplemented with 1% (v/v) Tween 80; C – MHA supplemented with 1% (v/v) Tween 80 and subinhibitory concentration of *trans*-anethole. ne – no expression.
